# Development of clinical teaching competency framework and scale for physician teachers in China: an exploratory sequential mixed methods study

**DOI:** 10.1080/10872981.2025.2599745

**Published:** 2025-12-09

**Authors:** Qin Chen, Fancai Zeng

**Affiliations:** a International Education School, Southwest Medical University, Luzhou, Sichuan, People's Republic of China; b Preclinical Medicine School, Southwest Medical University, Luzhou, Sichuan, People's Republic of China

**Keywords:** Clinical teaching competence, physician teachers, faculty development, competency assessment scale, good health and well-being

## Abstract

Physician teachers (PT) play a vital role in professional health education. However, there is a lack of a theoretical framework to direct PT faculty development in China. Therefore, this study was designed to develop and validate a clinical teaching competency framework and scale (CTCS) for physicians in China. This mixed-methods study began with qualitative interviews and a Delphi process to develop an initial framework and a pretest version of the Clinical Teaching Competency Scale (CTCS). Using convenience and random sampling, 450 physician teachers (PTs) were selected as the prediction sample. A national survey involving 3,179 PTs from seven administrative regions in Mainland China served as the validation sample. Exploratory and confirmatory factor analyses were conducted to assess construct validity. Seven dimensions were identified. Namely, intellectual literacy, clinical curriculum teaching competence, clinical practice teaching competence, role modeling, clinical teaching choice ability, clinical teaching emotional ability, and clinical teaching independent development ability. These explained 70.513% of the total variance. The final scale comprised 54 items, with a Cronbach’s alpha of 0.987. Confirmatory factor analysis showed acceptable fit indices: RMSEA = 0.065, SRMR = 0.038, and GFI, NFI, IFI, TLI, and CFI all > 0.9. Construct reliability (CR) for each dimension exceeded 0.9, and average variance extracted (AVE) ranged from 0.672 to 0.832, indicating strong reliability and validity. A pyramid model with seven domains and 54 items was developed using a mixed-methods approach. This framework can guide the training of physician teachersand ultimately support health and well-being. The CTCS demonstrates acceptable reliability and validity, making it a valuable tool for assessing the clinical teaching competence of physician teachers.

## Introduction

### The importance of clinical teaching

Clinical teaching is a core component of doctors’ professional responsibilities and plays a crucial role in developing the professional skills and practical knowledge of future physicians [1]. Physician-teachers, who are physicians with educational roles in either classroom or clinical settings, possess both medical qualifications and teaching credentials and support the growth of future healthcare professionals [2,3]. The importance and responsibility of physician teachers have been recognised since the era of the Hippocratic Oath [4]. However, this historical recognition contrasts with contemporary systemic challenges that undermine teaching efficacy. The guidelines issued by the World Health Organisation emphasise the importance of strengthening faculty development activities to produce competent and motivated doctors in the long term [5]. In this context, the role of Faculty Development Programmes (FDPs) in developing competent medical teachers is well acknowledged [6–8]. However, challenges faced by FDPs during the implementation of Competency-Based Medical Education (CBME) include a lack of guidance from educational theory and teaching competency frameworks, which limits their potential impact [9]. Hartford indicates that traditional approaches to medical teacher development have reduced teaching effectiveness [10], highlighting the need for reform in clinical teacher training. In high-pressure hospital environments, clinical duties often overshadow teaching responsibilities, fostering the belief that ‘I am a doctor and not a teacher [4,11–13].’ The undervaluing of teaching, unclear criteria, and unreliable metrics pose significant barriers to the recognition of clinician educators [14]. Typically, clinical tasks are prioritised over educational efforts [15]. Implementing a set of guidelines could support educators in developing the necessary skills to bridge this gap. Therefore, a comprehensive, evidence-based framework outlining the core teaching skills required by physician teachers and providing reliable assessment tools to support their ongoing professional development is urgently needed.

### Literature review

Competency-based medical education (CBME) is currently driving improvements in the training of physician teachers [16], with growing attention to the study of teaching competencies in medical education, particularly within the CBME framework. International bodies such as the UK General Medical Council (GMC) [17] and the US Accreditation Council for Graduate Medical Education (ACGME) [18] emphasise the critical role of instructional abilities in clinical teachers. The Association for Medical Education in Europe (AMEE) Guide No. 20 further delineates 12 key roles for teachers, including classroom lecturing, on-the-job role modelling, curriculum evaluation, curriculum planning, and course organisation [19,20]. Previous studies have characterised effective clinical teachers as possessing not only clinical expertise, compassion, and dedication to teaching, but also strong teaching motivation, integrity, and pedagogical skills [21,22]. Steinert highlighted the centrality of experiential learning in teacher competency development, categorising it into three modes: observational, practice-based, and reflective learning [23]. This aligns with the ‘Teaching as a Competency’ model, which—drawing on frameworks such as CanMEDS and ACGME—identifies six core competencies: medical content knowledge, learner-centeredness, interpersonal and communication skills, professionalism and role modelling, practice-based reflection, and systematic practice [24]. However, while research has explored diverse aspects of clinical education, including instructional strategies, teaching methodologies, and assessment approaches [25], integrating clinical teaching into physicians’ professional roles remains challenging, particularly in developing systematic and reliable methods for evaluating teaching skills [26]. Notably, progress has been made in establishing standards for educators in other contexts [27]. The UK Academy of Medical Educators has outlined proficiency levels that advance as educators develop their skills and assume greater responsibilities [28]. International competencies for nurse educators—encompassing clinical expertise, effective communication, and educational leadership—have been established to ensure that they are equipped to train the next generation of healthcare professionals [29]. This emphasis on continuous professional development underscores its importance in maintaining high standards of patient care and advancing the healthcare profession. However, a notable gap remains in recent research on workplace education for allied health professionals [30].

Despite the recognised importance of clinical teaching competencies, the existing literature reveals significant gaps in clinical teaching competencies framework and assessment tool for physician teachers in China. First, it lacks empirical support and validation within China’s clinical education context. Second, it often lacks an assessment tool for physician teachers' clinical teaching competencies and focuses on technical competence while neglecting non-technical competence.

To address these issues, this study designed an exploratory sequential mixed methods study to develop a clinical teaching competency framework and scale for physician teachers in China that could help with faculty development.

In the literature, the terms physician teacher and clinical teacher are sometimes used interchangeably. ‘Physician teacher’ in this study refers especially to certified medical professionals who have teaching duties, acknowledging their dual roles in clinical practice and medical education.

## Methods and participants

### Study design and methodology

**Figure 1.**
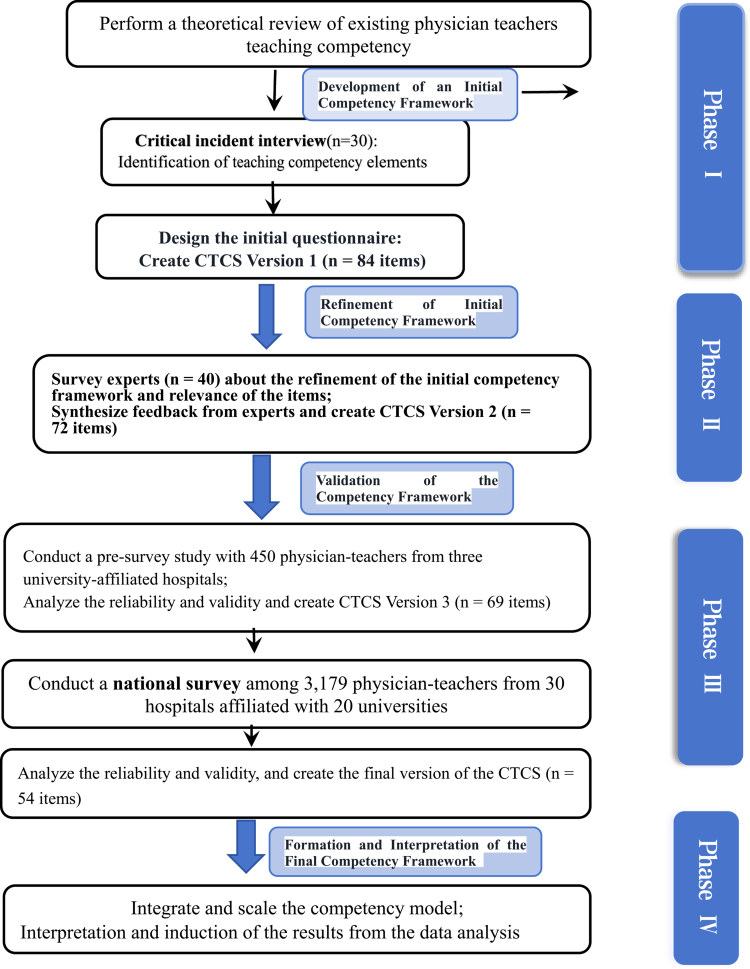
[31] Research study design.

This study utilised an exploratory sequential mixed-method design (see Figure 1) to develop a clinical teaching competency framework for physician teachers [31]. The methodology begins with qualitative research aimed at constructing a theoretical framework of teaching competence, which is subsequently validated through quantitative methods.

This mixed-methods approach is closely aligned with the study's objectives, as it reflects the emotional and humanistic dimensions inherent in traditional Chinese medicine culture. In this context, medicine embodies both uncertainty and the art of possibilities [32]. Thus, the humanistic aspect of medical education positions qualitative research as a primary paradigm for this study, emphasising the importance of individual experiences, complexity, and the specificity of various subjects [33]. Such characteristics highlight the necessity for a methodology adept at capturing the uniqueness of the educational landscape. Consequently, qualitative research is selected for its focus on theory construction based on real educational contexts. At the same time, recognising the need to quantify the theoretical model, the incorporation of quantitative research is deemed essential.

### Study participants

#### Interviewees

This study used qualitative methods to investigate physician teachers' perceptions and the structure of clinical teaching competencies. We selected participants using purposive sampling, which was guided by the principles of maximum variation and information saturation. The inclusion criteria were as follows: (1) a minimum of two years of clinical teaching experience with continuous medical course instruction; (2) possession of both a practicing physician's license and a university teacher's qualification certificate; and (3) willingness to engage in the study. The sample consisted of 30 physician teachers from 12 provinces in China, including Beijing, Jiangsu, Liaoning, Sichuan, Guangdong, Shandong, Ningxia, Fujian, Tianjin, Hunan, Shanghai, and Heilongjiang, with a gender distribution of 13 males and 17 females.

#### Expert panel

The Delphi technique is a systematic forecasting process that uses the collective opinions of panel members [34]. A panel of 40 experts was invited to a questionnaire consultation to assess and guide the improvement of the questionnaire items' structure, content, and expressions. The expert inclusion criteria were as follows:

(1) Geographic Distribution: Experts were selected from all seven major administrative regions of China (Northeast, North, Northwest, East, Central, South, and Southwest) through stratified sampling to ensure regionally representative.
(2) Authoritativeness and Comprehensiveness: The selection focused on five key categories: medical educators, physician teachers, public health experts, education scholars, and medical teaching faculty developers. An initial list of 97 experts was compiled based on their involvement in the past five years in activities such as national medical education forums, medical teacher development alliance conferences, debates, literature salons in medical education, and the FAIMER regional centre in China.
(3) Professional Experience: Researchers who have published influential papers in medical education and those who are dedicated to teacher development.

#### Questionnaire respondents

Subjects for the pre-test sampling were chosen from three university-affiliated institutions and three teaching hospitals in Sichuan Province, China, primarily focusing on physician teachers who are certified as university faculty and are actively engaged in the instruction of medical undergraduates. To ensure diverse representation, a simple random sampling method was employed, resulting in the distribution of 502 questionnaires, from which we received 450 valid responses, achieving an impressive 95.5% response rate.

For the formal survey, we utilised stratified sampling to account for various factors, including medical teaching quality, geographic location, institutional type, and affiliation. This approach encompassed 20 provinces in China, including Sichuan, Beijing, and Jiangsu, and we successfully collected 3,179 questionnaires from informed and consenting participants.

The dissemination of these questionnaires was conducted via the WJX platform for online replies, and they were additionally provided in print during faculty meetings and training sessions to optimise outreach and participation.

### Study phases

This study had four phases: (1) Qualitative interviews were used to develop a preliminary list of competencies for CTCS,and the identified components of PTs that guided the development of a preliminary questionnaire. (2) Delphi expert consultation refined these competencies and ensured content validity through expert consensus. (3) A pre-questionnaire study, followed by a national survey, validated and further refined the competency model, enhancing its applicability across settings. (4) The competency model is visually represented in the final framework, which makes the conclusions easier to understand for future research and practical applications.

#### Phase 1: qualitative interviews: initial clinical teaching competency framework and scale development for physician teachers

First, we conducted a semi-structured interview with 30 experienced physician teachers to investigate their understanding of clinical teaching competency. The interviews covered the following topics:

(1) What do you think are the essential competencies that physician teachers should possess in order to be successful in clinical teaching? Please provide examples.
(2) What are the most challenging problems encountered during teaching? How do you handle these situations? What are the outcomes of your approach, and why did you choose that particular approach?
(3) What are the three most satisfying experiences you experienced while teaching? How did you achieve success in these instances and why did you think you were so effective? How did others respond?
(4) What competencies should physician teachers possess to fulfil undergraduate clinical teaching tasks? Do you know of any universally recognised standards for clinical teaching competency?
(5) If you were to provide guidance to a newly appointed physician teacher, what would you focus on and how would you guide them?

Following each interview, the researcher transcribed the interview recordings verbatim and used a visible thinking analysis framework as a reference to help recall and describe the interviewees' language, actions, and facial expressions. Based on the coding results, a preliminary framework for clinical teaching competency was developed, which consisted of six dimensions, 17 sub-indicators, and 82 items. The initial questionnaire was compiled by using this framework.

#### Phase 2: two rounds of the Delphi consultation: refinement of the initial competency framework and establishment of the predictive questionnaire

To assess content validity, a Delphi expert consultation was conducted using a questionnaire. Forty experts were asked to assess whether the questionnaire variables adequately represented the study's concerns, whether each variable's dimension division made sense, whether each item's expression adhered to best practices, and whether each item conveyed its intended meaning accurately. Seven dimensions, 19 sub-indicators, and 72 items constituted the initial framework for clinical teaching competency that was created based on the experts' agreement on competencies. The framework was used to generate the first questionnaire.

This study developed a preliminary theoretical model for the teaching competency of physician instructors in Chinese universities after two rounds of expert consultations. Furthermore, a survey questionnaire was created to evaluate the components of teaching competency, which called for additional quantitative verification. Seven basic domains, 19 secondary indicators, and 72 questionnaire questions are included in the model.

#### Phase 3: quantitative research: verification of the preliminary framework

A two-step cross-sectional survey was used in this phase, consisting of a national survey of 3,179 physician teachers (PTs) and a preliminary survey of 450 PTs.

The purpose of this preliminary survey was to improve the questionnaire. A total of 501 PTs from our institution participated in a convenience sample pilot study in December 2022 to assess the CTCS for Chinese PTs; 450 valid responses (89.8% response rate) were obtained. The critical ratio was determined in the first analysis using independent sample T-tests in SPSS 22.0, with the criterion of a critical ratio greater than 3 and *p* < 0.05. We examined correlations between items and total scores, keeping items with correlations >0.4 and *p* < 0.05, if removing them did not raise the Cronbach's alpha of the scale. Principal components were found using exploratory factor analysis (EFA), with Bartlett's test *p* < 0.05 and Kaiser-Meyer-Olkin measure above 0.8. Items with eigenvalues greater than one and factor loads ≥0.50 were chosen using principal component analysis and varimax rotation. The final CTCS had 54 items, 19 sub-indicators, and seven dimensions.

A formal questionnaire was used in the nationwide survey to validate the teaching competency model and scale. With assistance from the CMU-FAIMER and Medical Education section of the Chinese Medical Association, 3,500 PTs in seven different regions of China received an improved CTCS between January and March 2023. This resulted in 3,179 valid responses with a response rate of 90.83%. A structural equation model and fit indices were employed for validation [35], and the values of GFI, AGFI, NFI, IFI, CFI > 0.90, PCFI and PNFI > 0.5, RMSEA < 0.1, and RMR < 0.05 showed an adequate model fit. Composite reliability (CR > 0.7) and average variance extracted (AVE > 0.5) were used to evaluate convergent validity, and the results showed strong convergent validity [36]. The analysis used Cronbach's alpha for internal consistency and Pearson's correlation for item-scale correlation, using SPSS 22.0. The internal structure of the scale was investigated using factor analysis, which included a principal component analysis and varimax rotation. AMOS 26.0 was used to perform confirmatory factor analysis, with a two-tailed significance level of *α* = 0.05.

#### Phase 4: development of the final framework and visual representation of the competency model

This phase involved the development and clarification of the final framework through the integration of qualitative and quantitative data analyses, culminating in the construction of a diagram representing the competency model. The main aim of this diagram is to offer a clear and succinct visual depiction of the links among different competences and their corresponding subcomponents. The competencies identified in the survey were initially classified into primary domains, each representing a specific facet of clinical teaching competency. Subsequently, the subcomponents within each domain were identified and systematically linked to their respective competencies, thus establishing a cohesive framework.

The diagram was evaluated by field specialists, who provided suggestions for enhancement to ensure correctness and efficacy. Adjustments were implemented based on feedback to improve the clarity and readability of the diagram. This iterative procedure sought to enhance the comprehension of the overarching competency model.

### Reflexivity and quality management

Multiple measures were used to ensure data reliability and validity. The interviews for the qualitative research component were performed by the authors, who got comprehensive training in both qualitative and quantitative research methodologies throughout their doctoral studies in education. Informed consent was secured from all participants, and specified criteria were implemented to ensure data neutrality and validity. Questionnaires that required less than five minutes or more than thirty minutes to complete, had consistent responses, or diverged from the specified instructions were omitted from the study. The purpose of the study was explicitly conveyed to the teachers, highlighting the significance of authentic feedback and stating that individual performance evaluations are not objective. The researchers assumed a learner's perspective during the interviews to reduce their effect on the participants, thus facilitating more empathic and impartial interactions. Reflective thinking was utilised to critically evaluate the acquired facts, ensuring significant interpretation and profound analysis. These quality management measures sought to maintain elevated standards of research integrity throughout the investigation.

## Results

### Demographical characteristics of the prediction samples and verification samples

The characteristics of the participants in both the prediction and verification versions of the survey are shown in Table 1.

**Table 1.** Demographic characteristics of physician teachers from 31 provinces.

Characteristics		Verification sample *N* = 3179	Prediction sample *N* = 450
Gender	Male	896	184
	Female	2283	266
Age	Below 30 years	314	2
	31-40years	1616	288
	41-50years	989	105
	51-60 years	252	55
Region	Northeast China	297	50
	Northern China	632	
	Eastern China	743	
	Southern China	737	46
	Central China	608	54
	Northwest China	55	50
	Southwest China	107	250
The title of a professional post	Primary	582	116
	Middle	1261	167
	Vice-senior	983	98
	senior	353	69
Academic degree	Bachelor	1197	82
	Master	1167	279
	Doctor	815	89

### Analysis of prediction samples

#### The results of the item and reliability analysis

revealed that the CR values varied from 6.031 to 18.809, with *p*-values below 0.05. The correlation coefficients between each item score and the total scale score varied from 0.575 to 0.780, with *p*-values below 0.05. The overall Cronbach's alpha coefficient was 0.953. Subsequently, item analysis and reliability analysis were performed for each level index. Following these analyses, items 36, 53, and 54 were removed from the questionnaire, yielding a revised scale of 69 questions.

#### Exploratory factor analysis

Table 2 was performed after the item and reliability analysis, resulting in the removal of 3 items. Factor analysis was conducted on the remaining 69 items to identify underlying components. A sampling adequacy assessment was performed, resulting in a Kaiser-Meyer-Olkin value of 0.953 and a significant Bartlett's test of sphericity (χ^2 = 25587.169, df = 1540, *p* < 0.001), confirming the suitability of exploratory factor analysis for the scale.

**Table 2.** Exploratory factor analysis of the clinical teaching competence scale.

	Factor load coefficient							
Item	KC	ZZ	QG	SZ	YJ	ZS	SJ	Communality
5	0.586							0.626
6	0.549							0.665
7	0.619							0.676
8	0.589							0.660
9	0.687							0.656
10	0.651							0.634
11	0.681							0.649
12	0.739							0.662
13	0.674							0.676
14	0.593							0.503
15	0.723							0.733
16	0.663							0.699
52		0.590						0.606
64		0.636						0.681
65		0.711						0.771
66		0.676						0.772
67		0.758						0.782
68		0.728						0.796
69		0.743						0.813
70		0.739						0.796
71		0.725						0.780
72		0.705						0.769
39			0.737					0.727
40			0.751					0.773
41			0.609					0.680
46			0.725					0.728
48			0.751					0.777
50			0.589					0.662
58			0.666					0.683
62			0.613					0.685
63			0.647					0.732
27				0.554				0.642
28				0.598				0.711
29				0.620				0.707
30				0.617				0.746
31				0.674				0.793
32				0.678				0.732
33				0.552				0.652
34				0.486				0.651
35				0.574				0.611
37				0.690				0.692
38				0.681				0.724
17					0.639			0.657
18					0.599			0.722
19					0.719			0.762
20					0.748			0.794
21					0.614			0.719
1						0.662		0.646
2						0.768		0.730
3						0.731		0.723
4						0.551		0.568
22							0.741	0.812
23							0.686	0.768
24							0.575	0.727
Eigenvalues Before Rotation▯	26.775	4.331	2.412	2.114	1.419	1.227	1.209	
Cumulative Percent of Variance Explained (After Rotation)▯	14.349%	28.330%	40.664%	52.982%	59.400%	65.024%	70.513%	

Abbreviations: KC = Clinical course teaching ability; ZZ = Self-development competence; QG = Personal traits and emotional competence; SZ = Role modelling and suitability for clinical teaching; SJ = Clinical practice teaching ability; YJ = Clinical teaching research skills; ZS = Clinical knowledge literacy.

Exploratory factor analysis was conducted using principal component analysis with oblique rotation (oblimin). Factors were retained according to eigenvalues exceeding 1, total variance accounted for, and the scree plot analysis. Items exhibiting communalities below 0.50, factor loadings below 0.45, or cross-loadings beyond 0.4 were deemed eligible for removal.

During the exploratory process, it was observed that several items deviated significantly from their corresponding factors, and one item loaded on multiple factors. In accordance with the item deletion criteria, 15 items were removed, yielding a final total of 54 items. Seven significant factors were identified, explaining 70.513% of the variance. The factor structure demonstrated clarity and logical consistency, with item loadings ranging from 0.458 to 0.768.

### Analyses of the validation sample

Analysis of the validation sample revealed good discrimination, high reliability, and validity of the formal questionnaire. Item analysis was conducted on all items using the critical ratio method to test score differences between the high- and low-score groups for clinical teaching competence across 54 items. Items showing significant differences were retained, while those lacking significant differences were subjected to expert panel discussions or correlation analysis to ascertain whether to eliminate or amend them. The results revealed substantial disparities across all 54 items, with correlation values exceeding 0.4, indicating strong discriminative power for each item (refer to Table 3).

**Table 3.** Item analysis results of the questionnaire assessing clinical teaching competence of physician teachers.

	Group (mean ± standard deviation)			
Item	Low score group (*n* = 1240)	High score group (*n* = 880)	t (critical value)▯	Correlation with the total score on the scale
1	4.56 ± 1.16	5.64 ± 0.62	27.77	0.629**
2	4.51 ± 1.19	5.69 ± 0.58	30.208	0.650**
3	4.49 ± 1.19	5.70 ± 0.59	31.042	0.663**
4	4.27 ± 1.23	5.70 ± 0.74	33.439	0.649**
5	4.16 ± 1.19	5.81 ± 0.62	41.597	0.730**
6	4.20 ± 1.20	5.86 ± 0.47	44.257	0.759**
7	4.16 ± 1.19	5.87 ± 0.47	45.699	0.780**
8	4.16 ± 1.20	5.90 ± 0.41	47.278	0.779**
9	4.07 ± 1.18	5.85 ± 0.59	45.503	0.735**
10	4.21 ± 1.18	5.91 ± 0.39	47.297	0.797**
11	4.10 ± 1.20	5.84 ± 0.63	43.203	0.741**
12	3.98 ± 1.19	5.79 ± 0.72	43.44	0.700**
13	4.08 ± 1.19	5.93 ± 0.40	50.838	0.766**
14	3.99 ± 1.22	5.83 ± 0.63	44.961	0.717**
15	3.99 ± 1.18	5.90 ± 0.47	51.622	0.755**
16	4.02 ± 1.17	5.90 ± 0.46	51.442	0.768**
17	4.04 ± 1.18	5.77 ± 0.78	40.581	0.683**
18	4.07 ± 1.18	5.87 ± 0.58	46.155	0.730**
19	3.98 ± 1.19	5.79 ± 0.74	43.091	0.705**
20	3.97 ± 1.20	5.80 ± 0.72	43.614	0.716**
21	4.01 ± 1.17	5.88 ± 0.56	49.001	0.760**
22	4.13 ± 1.19	5.86 ± 0.58	44.264	0.759**
23	4.18 ± 1.19	5.86 ± 0.57	43.135	0.762**
24	4.14 ± 1.18	5.90 ± 0.48	47.372	0.781**
25	4.25 ± 1.17	5.94 ± 0.29	49.072	0.824**
26	4.21 ± 1.17	5.93 ± 0.37	48.243	0.814**
27	4.28 ± 1.15	5.97 ± 0.18	50.762	0.838**
28	4.30 ± 1.14	5.95 ± 0.26	48.95	0.839**
29	4.41 ± 1.13	5.92 ± 0.29	45.145	0.823**
30	4.35 ± 1.15	5.95 ± 0.26	47.502	0.840**
31	4.31 ± 1.15	5.96 ± 0.24	48.855	0.840**
32	4.29 ± 1.17	5.97 ± 0.18	49.805	0.837**
33	4.34 ± 1.15	5.94 ± 0.32	46.335	0.824**
34	4.35 ± 1.14	5.94 ± 0.28	47.222	0.819**
35	4.40 ± 1.14	5.94 ± 0.25	46.29	0.828**
36	4.58 ± 1.07	5.78 ± 0.42	35.852	0.773**
37	4.58 ± 1.10	5.79 ± 0.44	35.114	0.765**
38	4.50 ± 1.14	5.87 ± 0.37	39.353	0.806**
39	4.54 ± 1.12	5.79 ± 0.41	36.048	0.786**
40	4.57 ± 1.11	5.80 ± 0.40	35.973	0.793**
41	4.55 ± 1.11	5.81 ± 0.41	36.758	0.788**
42	4.58 ± 1.09	5.79 ± 0.41	35.693	0.788**
43	4.57 ± 1.10	5.79 ± 0.41	35.609	0.788**
44	4.55 ± 1.11	5.80 ± 0.41	36.283	0.789**
45	4.42 ± 1.17	5.89 ± 0.38	41.184	0.792**
46	4.45 ± 1.16	5.89 ± 0.34	41.395	0.810**
47	4.45 ± 1.15	5.90 ± 0.31	42.069	0.823**
48	4.38 ± 1.18	5.89 ± 0.40	42.096	0.791**
49	4.33 ± 1.17	5.90 ± 0.39	43.59	0.809**
50	4.36 ± 1.16	5.90 ± 0.37	43.739	0.819**
51	4.35 ± 1.16	5.89 ± 0.37	43.971	0.812**
52	4.40 ± 1.16	5.92 ± 0.31	43.882	0.815**
53	4.34 ± 1.17	5.91 ± 0.34	44.598	0.809**
54	4.33 ± 1.18	5.92 ± 0.31	45.367	0.805**

** *p* < 0.0001.

The reliability analysis (Table 4) showed an internal consistency coefficient of 0.987 for the entire Clinical Teaching Competence Questionnaire, with primary dimensions displaying reliability coefficients exceeding 0.9. The composite reliability for the seven dimensions exceeded 0.9, while the average variance extractions surpassed 0.5, indicating high reliability and convergent validity.

**Table 4.** Internal consistency reliability and convergence validity of the questionnaire.

Domain	Cronbach's Alpha	CR	AVE
Clinical knowledge literacy	0.904	0.906	0.708
Clinical course teaching ability	0.961	0.961	0.672
Clinical teaching research skills	0.943	0.944	0.770
Clinical practice teaching ability	0.931	0.932	0.820
Role modelling and suitability for clinical teaching	0.977	0.977	0.797
Personal traits and emotional competence	0.978	0.978	0.832
Self-development competence	0.978	0.974	0.788
All	0.987		

Discriminant validity analysis (Table 5) was conducted by comparing the square root values of the average variance extraction (AVE) for the seven factors with correlation coefficients. The results demonstrated that the AVE square root values were larger than the correlation coefficients, indicating good discriminant validity among the factors.

**Table 5.** Differential validity of teaching competence model for physician teachers.

Distinguishing validity: Pearson correlation and AVE square root values							
	Clinical knowledge literacy	Clinical course teaching ability	Clinical teaching research skills	Clinical practice teaching ability	Role modelling and suitability	Personal traits and emotional competence	Self-development competence
Clinical knowledge literacy	0.842						
Clinical course teaching ability	0.669	0.82					
Clinical teaching research skills	0.530	0.756	0.878				
Clinical practice teaching ability	0.562	0.738	0.665	0.905			
Role modelling and suitability	0.610	0.746	0.678	0.769	0.893		
Personal traits and emotional competence	0.596	0.633	0.55	0.61	0.788	0.912	
Self-development competence	0.605	0.721	0.617	0.675	0.8	0.829	0.888

Note: Diagonal blue numbers are AVE square root values.

The model fit of the theoretical model of clinical teacher teaching competency was tested (Table 6). Tests for skewness and kurtosis confirmed the normal distribution of the data. The model fit indices met the criteria for a good fit: Root Mean Square Residual (RMSR) = 0.065, Standardised Root Mean Square Residual (SRMR) = 0.038, Goodness-of-Fit Index (GFI) = 0.92, Normed Fit Index (NFI) = 0.91, Incremental Fit Index (IFI) = 0.918, Tucker-Lewis Index (TLI) = 0.913, and Comparative Fit Index (CFI) = 0.92. Furthermore, two alternative models were compared, and the first-order seven-factor oblique Model C demonstrated the optimal match with the data.The analysis of the validation sample confirmed the good discrimination, high reliability, and validity of the formal questionnaire. The theoretical model of clinical teacher teaching competency demonstrated a good fit with the empirical data.

**Table 6.** Fit indices for confirmatory factor analysis of the clinical teaching competency model.

Thresholds for fit indices	RMSEA	RMR	CFI	NFI	NNFI	TLI	IFI	SRMR
Fit indices	<0.10	<0.05	0.9	0.9	0.9	0.9	0.9	<0.1
Model C	0.065	0.047	0.92	0.91	0.91	0.913	0.918	0.038
Model Ct1	0.072	0.056	0.901	0.895	0.896	0.896	0.901	0.043
Model Ct2	0.082	0.083	0.870	0.865	0.864	0.864	0.870	0.067

### A diagram of the clinical teaching competency model for physician teachers

**Figure 2.**
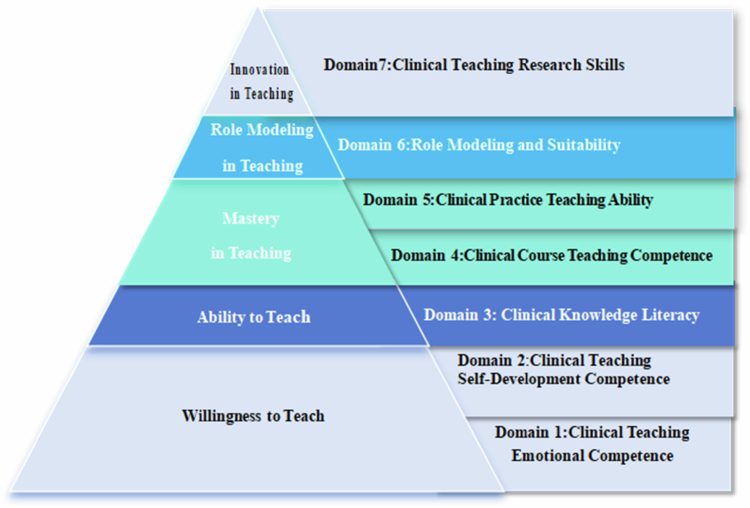
Clinical teaching competency pyramid model for physician teachers in China.

We used the above analysis results and ultimately constructed a general teaching competency model for physician teachers in china (as illustrated in Figure 2).

The structural model of physician teachers' clinical teaching competency is depicted as a golden pyramid, encompassing seven dimensions: (1) clinical teaching emotional ability, (2) clinical teaching independent development ability, (3) intellectual literacy, (4) clinical curriculum teaching competence, (5) clinical practice teaching ability, (6) role modelling, and (7) clinical teaching research ability. These dimensions are further categorised as follows: the Emotional Ability domain includes professional traits and moral character development; the Independent Development Ability domain covers teaching growth, exchange, and cooperation; the Intellectual Literacy domain consists of disciplinary knowledge and general education; the Curriculum Teaching Competence domain is divided into curriculum design, teaching implementation, and evaluation; the Clinical Practice Teaching Ability domain comprises ward rounds, bedside teaching, and case discussions; the Role Modelling domain includes role-building, clinical teaching selection, and student feedback; and the Teaching Research Ability domain encompasses clinical teaching knowledge production and evidence-based teaching.

The pyramid model consists of five stages. The first stage, ‘​​​​​​**Willingness to Teach’** represents the initial motivation to engage in clinical teaching. This stage begins with the development of ‘clinical teaching emotional ability’ and ‘clinical teaching independent development ability.’ Physicians at this stage must balance their roles as both teachers and clinicians, focusing on developing their clinical teaching skills. However, if they merely acknowledge their teaching identity verbally or remain at a superficial level of autonomous development without actionable growth, they will remain stalled at this stage. Only by recognising their teaching identity and actively engaging in clinical teaching development can they progress to the second stage, ‘Ability to Teach.’ The second stage, ‘​​​​​​**Ability to Teach’** represents the foundational knowledge required for clinical teaching. Clinical teachers must possess robust intellectual literacy, including disciplinary knowledge and general education. With this foundation, they can advance to the third stage, ‘​​​​​**Mastery in Teaching’** where they acquire essential teaching abilities such as ‘clinical course teaching competence’ and ‘clinical practice teaching capability.’ Mastery of these skills enables them to deliver instruction confidently.The fourth stage, ‘​​​​​​**Role Modelling in Teaching**’ signifies the importance of exemplary behaviour and clinical teaching decision-making. Educators at this stage excel at ‘teaching by example,’ recognising that their every action in front of medical students and patients carries both educational and clinical significance. Unique competencies such as role-building, clinical teaching selection, and student feedback distinguish physician teachersfrom their counterparts in other academic disciplines. At the pinnacle of the pyramid, the fifth stage, ‘​​​​​​**Innovation in Teaching’** represents the highest level of clinical teaching. Educators at this stage engage deeply in clinical settings, identifying and leveraging the latest clinical knowledge to transform it into symbolic knowledge. This includes the production of clinical knowledge and evidence-based teaching practices. These educators enjoy exploring new clinical insights with their students, applying the best evidence-based principles in instruction. Compared with other physician educators, they possess a deeper understanding and unique perspective on the significance and value of clinical teaching.

## Discussion

### Comparison with other competency frameworks

Our study addressed specific inadequacies in China's clinical teacher training system by creating a model with seven domains and 19 secondary indicators. in contrast to previous competency frameworks [1,17,18,20,22,24,37], ours integrates often-overlooked dimensions such as role modelling, innovative teaching, and clinical emotional competence within China's distinctive educational context [38,39]. The unique features of the framework address critical gaps in three aspects.First, it aligns with China's national teacher certification requirements [40] while resolving the current imbalance that overemphasises technical skills [40]. Second, while prior research has identified teacher willingness as crucial [41,42], China’s physician teacher training has traditionally overemphasised technical skills at the expense of emotional competence and role modelling [40]. Our framework specifically addresses this gap by validating emotional competence as critical for effective teaching [42,43] and informing the teaching hospital recruitment criteria [44]. Third, the inclusion of role modelling aligns with Molodysky's conceptualisation of physician teachers [45] and Bandura's social learning theory [46], It also addresses the current gap wherein most clinical teachers lack formal training [22], leaving informal role modelling (through hidden curricula) influential yet unregulated [47]. Our framework makes this implicit process measurable and actionable. Finally, the teaching research ability domain reflects the evolving scholar-educator paradigm [48,49], while the clinical practice teaching component bridges Osler's patient-centred tradition with modern evidence-based practice [50].

In summary, our framework seeks to contribute by integrating cultural values, addressing training gaps, and engaging with global theory. It is our hope that this research may enhance applicability to China’s clinical education landscape and offer a modest reference for balancing cultural relevance with evidence-based practice in medical education globally.

### Developing the pyramid model of clinical teaching competency

This study adopted an exploratory sequential mixed-methods approach across most china’s provinces to identify the teaching competencies of physician teachers. It develops a pyramid model to guide physician-teacher instruction, offering insights for both theoretical development and practical application.

Prior studies note the ambiguity of medical competence [51,52]; this research clarifies the concept within medical education by defining clinical teaching competency as a multifaceted and evolving construct. It encompasses the competencies required to instruct medical students, manage interactions with students, patients and the external environment, fulfil educational responsibilities, and advance medical education goals. This enhances understanding of competence and its use in competency-based medical education [53].

### A model demonstrating five tiers of progressive unique characteristics

The pyramid model outlines five progressive tiers of clinical teaching competency: willingness to teach, ability to teach, mastery in teaching, role modelling in teaching, and innovation in teaching. Though physicians are expected to teach [54], time constraints make willingness to teach indispensable [55]. These tiers reflect competence progression and element interrelation, acknowledging China’s emotional priorities and cultural context that influence competency development. Faculty development can drive such change [56], and the model must adapt to evolving healthcare practices and diseases.

### Formulating a theoretical framework for clinical teaching competence in china: a four-phase research model

This study presents an advanced four-phase methodology to establish a China-tailored theoretical framework for clinical teaching competency, accommodating the uniqueness of China’s educational and clinical contexts. The methodology includes interviews, expert consultation, pre-verification, and formal verification, with the final framework’s reliability and validity ensured through comprehensive data collection and analysis. The framework is grounded in empirical evidence, aligning with China’s unique educational, cultural, and clinical realities.

### CTCS exhibits high reliability and validity, along with significant practical value

The CTCS is highly reliable, valid, and practically valuable. Its standardisation provides a tool to evaluate clinical teaching competencies in healthcare settings, enabling objective assessment, quality improvement, customised development programmes for medical educators, and more accurate competency assessments—supporting physician-teachers’ continuous professional development.

## Limitations of the study and suggestions for further research

However, this study had several limitations. First, the sample’s diversity was limited, it may have a certain impact on the comprehensiveness of the research conclusions. Future research could expand the coverage of the survey and further test its implementation. Second, while the survey was conducted anonymously to encourage candid responses, the study remains subject to potential self-reporting biases inherent to this data collection method.

## Conclusion and future directions

Research has stressed the importance of theory and conceptual frameworks in physician-teacher training [56–58]. Seven basic domains, 19 secondary indicators, and 54 items comprise the Chinese physician teacher clinical competency pyramid model. Clinical teaching emotional capacity is central to the concept; doctors must teach by example, a unique skill. The model is culturally relevant and suited to China's medical education [59].

The 54-item Clinical Teaching Competency Scale (CTCS) has been found to be reliable and valid for Chinese physician teachers.The scale helps medical education progress by assessing present skills and finding areas for improvement.

Clinical teachers' teaching competency models are both a response to real problems and a search for the law of physician teachers' teaching development. They provide a new perspective on physicians' teaching development, theoretical support for solving the problem of insufficient specialisation in clinical teaching development, and help education management departments, universities, and hospitals provide a stronger guarantee.

## Data Availability

The datasets used and analysed during the current study are available from the corresponding author upon reasonable request.
